# Young men’s barriers to and facilitators of utilising HIV-testing services in South Africa

**DOI:** 10.4102/sajhivmed.v26i1.1631

**Published:** 2025-01-30

**Authors:** Sithembiso M.S. Ndlovu, Andrew Ross, James Ndirangu

**Affiliations:** 1 Department of Family Medicine, College of Health Sciences, University of KwaZulu-Natal, Durban, South Africa; 2 Office of the Dean of Health Sciences, Faculty of Health Sciences, University of the Free State, Bloemfontein, South Africa; 3 UNAIDS Country Office, Pretoria, South Africa

**Keywords:** HIV-testing services, young men, experiences, rural, peri-urban, South Africa, Ladysmith

## Abstract

**Background:**

In South Africa, men are less likely than women to use HIV-testing services (HTS). They are also unlikely to start and adhere to antiretroviral therapy until the virus has progressed to advanced AIDS stages.

**Objectives:**

To explore young men’s barriers to and facilitators of accessing and utilising HTS at the rural Driefontein and peri-urban Steadville Township in Ladysmith, KwaZulu-Natal (KZN) province, and to develop a comprehensive framework of care for young men to encourage and support them to utilise HTS at primary healthcare facilities.

**Method:**

This exploratory-descriptive qualitative study entailed using semi-structured interviews conducted via WhatsApp and landline audio calls with 17 young men between 18 years and 30 years of age in Steadville and Driefontein communities in KZN in September 2021. Participants were purposively and conveniently sampled, and the data were analysed thematically.

**Results:**

All participants were unmarried isiZulu African men experienced with HTS in the last 4 years. Fear of an HIV-positive test result, limited HTS knowledge, and stigma around HIV and AIDS were challenges linked to HTS utilisation. Unsafe sexual encounters, voluntary medical male circumcision, early virus-detection, having a significant other living with HIV, and HIV-status curiosity encouraged young men to utilise HTS.

**Conclusion:**

Various barriers and facilitators to HTS utilisation, are key for consideration when deriving contextual interventions acceptable to young men as efforts to raise awareness and attract and retain men in care.

**What this study adds:** The study contributes to knowledge about young men’s barriers and facilitators to using HTS in underserved communities to potentially improve their quality of life.

## Introduction

South Africa (SA) has the most extensive HIV antiretroviral therapy (ART) programme globally, which provides treatment to over 5.9 million individuals living with HIV.^1^ HIV testing remains a set point of entry into HIV care through various means, including diagnosis, linkage to care and, ultimately, viral load suppression.^2^ KwaZulu-Natal (KZN) province remains the province with the highest prevalence of HIV infections in SA.^3^

In SA, men living with HIV (MLHIV) remain underserved in the HIV care continuum,^4^ with men having worse HIV-related outcomes than their female counterparts.^5^ By 2019, approximately 2.8 million men over the age of 15 years were living with HIV in SA,^6^ with only 78% of South African men aware of their HIV-positive status compared with 89% of women.^5^ By 2021, over 8.2 million people in SA were living with HIV.^7^ A factor contributing to the high number of people living with HIV (PLHIV) is the lack of testing of men, which has been attributed to several factors, including fear of testing, fear of testing positive for HIV, and stigma associated with HIV/AIDS.^8,9,10,11,12^ In addition to the gaps in the HIV services provided, youth remain at high risk, as they are more susceptible to new HIV infection.^13,14^ Despite nationwide programmes and interventions to curb the spread of HIV among the youth through the inception of youth-friendly health services in healthcare facilities, there is still a challenge regarding their uptake of HIV-testing services (HTS).^15^ By 2017, only 67% of South African youth had accessed and used HTS.^3,16^

HIV-testing is an important step towards knowing one’s HIV status or how to prevent getting HIV if one tests negative.^17^ With most of the literature reporting on adolescent girls and young women’s acquisition of HIV,^18^ little is known about men’s HIV acquisition.^5^ A large body of literature has found that because women have a set entry point to care when pregnant, during which time testing for HIV is mandatory, more women test for HIV than men.^8,9^ Men are less likely to start and adhere to ART until the disease is advanced, which makes it a challenging task to achieve high rates of viral suppression and full immune recovery among men.^3,19^ Male HIV-related deaths often occur as a result of men not having sought HIV treatment, or delaying treatment access.^20^ The HIV treatment and care programmes benefit men and boys disproportionately less than women and girls, with men who have HIV having less-favourable health outcomes and being less likely to engage in HIV treatment and care services than women.^20^

Little attention has been paid to the psychosocial and structural factors that influence men’s access to HIV prevention and treatment programmes.^21^ An accurate understanding of HIV transmission, perceptions of HIV infection risks, attitudes and views of HTS, and previous experiences with HIV testing are all factors that impact testing decisions and practices.^1^ It has also been suggested that masculine gender norms contribute to men’s vulnerability to HIV, possibly because of expectations, in some cultures, for men to have multiple sexual partners.^5^ Furthermore, the stigma associated with HTS, long waiting times at health facilities, and the perception of a lack of confidentiality impede the uptake of these services.^22,23^ Use of HTS is also hampered by limited knowledge about HIV, fear of being infected, or confidentiality breaches.^22,24^

While studies have explored men’s barriers to testing and initiating HIV treatment in SA, current service delivery models are failing to reach sufficient men to reduce HIV prevalence and incidence in some parts of SA.^5^ There remains a need to identify and address barriers to HTS, which, if addressed, have the potential to enhance men’s health and quality of life, lower community viral load, and reduce HIV transmission to their sexual partners. Addressing these barriers to HTS could contribute to creating a stronger, more robust healthcare system in SA,^5^ in which men participate in HIV risk-reduction programmes prior to or at the onset of adulthood.^9^ The authors targeted young men for this, as HIV-prevention programmes seldom target men below 25 years of age. This research, which was part of a larger PhD study,^25,26^ aimed to determine what motivates and discourages young men in Steadville and Driefontein accessing and utilising HTS.

## Research methods and design

### Study design

This study used an exploratory-descriptive qualitative study design, with qualitative data collection methods, to explore and describe young men’s perceptions, knowledge, and experiences concerning their utilisation of HTS in Ladysmith, KZN province, SA.

### Setting and positionality

The study was conducted in the rural Driefontein and peri-urban Steadville Township communities, which are located 3 km and 30 km, respectively, from the town centre of Ladysmith in the uThukela District in KZN province, SA. By 2011, Steadville Township had a population of around 25 614, made up of mostly women (53%).^27^ The township population is mainly made up of black Africans (99.2%), with most of them speaking isiZulu (91.2%).^27^ Additionally, about 8500 of the township residents were aged between 0 years and 4 years (12.15%), 20 years and 24 years (11.15%), and 25 years and 29 years (10.86%).^27^ Driefontein had about 6774 residents by 2011, most of whom were black (99.9%), with about 96.5% of them speaking isiZulu.^28^ The majority of the population were women (53%).^28^ The two identified peri-urban and rural townships have a gap in the literature covering this subset of the population.

### Study population and sampling strategy

Seventeen young men were purposively (peri-urban Steadville participants) and conveniently (rural Driefontein participants) sampled to participate in the study. To be eligible to participate, they had to be 18 years to 35 years of age, provide informed consent, have no close relationship with the researcher, and have access to and be able to use the WhatsApp communication platform. Previous HTS experience was not a prerequisite for participation.

The principal researcher circulated a poster on WhatsApp and Facebook communication channels inviting young men in Steadville to participate in a qualitative study on their experiences and attitudes towards and use of HTS. The poster included the researcher’s contact information, with those who were interested contacting him directly via WhatsApp, and a date and time for the interviews scheduled. Eleven residents from Steadville were recruited via WhatsApp and Facebook communication channels.

As the principal researcher did not live in Driefontein, he posted the recruitment poster on one of the Driefontein community Facebook groups, but without any success. As a result, the recruitment poster was then sent to a colleague who assists young men in a HIV programme in Ladysmith. In order to comply with the *Protection of Personal Information Act (POPIA)*, No. 4 of 2013,^29,30,31,32^ the colleague, because of his involvement with the HIV programme, contacted young men who lived in Driefontein, briefed them on the research project, and asked if they were willing to participate in the study. Thirteen young men from Driefontein consented for him to share their names and contact details with the principal researcher, S.M.S.N., of whom six met the inclusion criteria and participated in the interviews at a time that was mutually convenient.

### Data collection

Semi-structured interviews (SSIs) were conducted with the 17 young men through the WhatsApp communication platform and landline audio calls in September 2021. Data collection was terminated at the 17th participant following data saturation. These data collection methods were chosen because of COVID-19 pandemic restrictions, which limited mobility and the ability to conduct face-to-face interviews. The WhatsApp communication platform and landline audio calls ensured adequate social distancing during the research process, thereby preventing COVID-19 transmission among the participants. The interviews lasted between 24 min and 72 min, were conducted in isiZulu, which was the primary language of the participants, and were audio recorded for later transcription and translation.

The interview guide contained open-ended questions that were informed by the integrated model of behaviour prediction, which was the guiding conceptual framework for the PhD study. The questions allowed the researcher to explore participants’ views, knowledge, beliefs, practices, and experiences relating to HTS in primary healthcare facilities, and their recommendations about how to improve the services and how to improve access to them for those who were reluctant. During the interviews, the researcher probed further as necessary and sought clarity on the responses by summarising what the participants had shared to ensure the correct data were captured.

Two pilot interviews with one participant from each community were conducted in order to ensure that appropriate data were collected. Thereafter, minor revisions were made to the interview guide by combining questions that overlapped and adding others that came up when the researcher probed further. Because of the minimal changes made to the questions, both interviews were included in the final analysis.

### Data analysis

All interviews were recorded and transcribed verbatim into isiZulu, after which they were translated into English by the principal researcher and then back translated to ensure accuracy of the translation. Thematic analysis was done in English, this approach being used to analyse the findings through Braun and Clarke’s^33,34^ six steps of data analysis, which are described in detail in other publications emanating from the PhD study.^25,26^

### Ensuring trustworthiness

Credibility of the findings was ensured by audio recording all the interviews, transcribing them verbatim and then translating and back translating the transcripts. Dependability was attained by taking participants through the research process and engaging with the findings in detail, to establish their consistency. To ensure transferability, a ‘thick’ description has been provided of the context, selection criteria and participants as well as the process of analysis. Confirmability was achieved by S.M.S.N. being cognisant of any potential bias and reflecting on any preconceived narratives that may have impacted the study with two other researchers (S.M.S.N., J.N.), who reviewed the research findings to ensure they minimised bias. Furthermore, the researcher, being from Steadville Township himself, took care to ensure that he was objective and unbiased throughout the research process.

### Ethical considerations

Ethical permission for the study was given by the Humanities and Social Sciences Research Ethics Committee (HSSREC) of the University of KwaZulu-Natal (UKZN) (reference no.: HSSREC/00000588/2019). As a result of COVID-19 and the restrictions imposed on data collection during the hard lockdown in South Africa in 2021, the researcher applied to the Research Ethics Committee for permission to conduct semi-structured interviews through WhatsApp communication platform instead of in person. This amendment was approved, and the researcher received updated ethical permission to change his data collection methods in July 2021.

Given that data were collected virtually, informed oral consent was obtained from all participants. To ensure confidentiality and anonymity, unique identifiers were allocated for every participant. Additional details are contained in other publications that emanated from the PhD study.^25,26^

## Results

### Research participants’ characteristics

Seventeen young, unmarried, isiZulu men, aged 18–30 years, whose education ranged from Grade 10 to postgraduate were interviewed for this research study (Table 1); all of the participants had experiences with HTS, with most having utilised both facility- and community-based testing facilities.

**TABLE 1:** Characteristics of study participants.

Alias	Age (years)	Race	Home language	Highest level of education	Marital status	Employment status	Location
P1	30	Black African	isiZulu	Postgraduate	Unmarried	Employed	Steadville
P2	30	Black African	isiZulu	Higher Certificate	Unmarried	Self-employed	Steadville
P3	20	Black African	isiZulu	Grade 12 (university student)	Unmarried	Unemployed	Steadville
P4	26	Black African	isiZulu	Grade 12 (university student)	Unmarried	Unemployed	Steadville
P5	29	Black African	isiZulu	Grade 12	Unmarried	Unemployed	Steadville
P6	21	Black African	isiZulu	Grade 10 (high school pupil)	Unmarried	Unemployed	Steadville
P7	22	Black African	isiZulu	Grade 12 (college student)	Unmarried	Unemployed	Steadville
P8	22	Black African	isiZulu	Bachelor’s degree	Unmarried	Employed	Steadville
P9	29	Black African	isiZulu	Grade 12	Unmarried	Employed	Steadville
P10	26	Black African	isiZulu	Diploma	Unmarried	Unemployed	Steadville
P11	28	Black African	isiZulu	Grade 12	Unmarried	Employed	Driefontein
P12	25	Black African	isiZulu	Grade 12	Unmarried	Self-employed	Steadville
P13	21	Black African	isiZulu	Grade 11 (high school pupil)	Unmarried	Self-employed	Driefontein
P14	25	Black African	isiZulu	Grade 12	Unmarried	Unemployed	Driefontein
P15	27	Black African	isiZulu	Grade 12	Unmarried	Employed	Driefontein
P16	26	Black African	isiZulu	Grade 12	Unmarried	Employed	Driefontein
P17	18	Black African	isiZulu	Grade 11 (high school pupil)	Unmarried	Unemployed	Driefontein

*Source*: Ndlovu S, Ross A, Mulondo M. Interventions to improve young men’s utilisation of HIV-testing services in KwaZulu-Natal, South Africa: Perspectives of young men and health care providers. Afr J AIDS Res. 2023;22(4):316–326. https://doi.org/10.2989/16085906.2023.2276897

### Key themes

The two themes relevant to HTS that emerged from the data analysis were (1) challenges linked to HTS utilisation, and (2) motivators to HTS utilisation. The challenges to HTS utilisation consisted of three sub-themes: fear of an HIV-positive test result, limited HTS knowledge, and stigma around HIV/AIDS. Five sub-themes emerged as motivators to HTS utilisation by young men: (1) engaging in unsafe sexual intercourse, (2) undergoing voluntary medical male circumcision (VMMC), (3) early HIV detection, (4) awareness of HIV status, and (5) having a significant other living with HIV (Table 2).

**TABLE 2:** Themes and sub-themes.

Theme	Sub-theme
1. Challenges to HTS utilisation	1.1 Fear of an HIV-positive test result 1.2 Limited HIV and HTS knowledge 1.3 Stigma around HIV/AIDS
2. Motivators to HTS utilisation	2.1 Engaging in unsafe sexual intercourse 2.2 Undergoing voluntary medical male circumcision 2.3 Early HIV detection 2.4 Awareness of HIV status 2.5 Having a significant other living with HIV

HTS, HIV-testing services.

#### Theme 1: Challenges to HIV-testing services utilisation

The research findings revealed that most participants reported constraints to accessing and using HTS, although they had all previously tested for HIV. In this regard, young men explained that they were scared to test for HIV, and cited the lack of information as a challenge to testing.

**Sub-theme 1.1: Fear of an HIV-positive test result:** Participants feared testing for HIV for a variety of reasons, mainly because of the possibility and implications of testing HIV positive following risky sexual encounters. They also reported their perceptions on the reasons why other young men do not utilise HTS:

‘I am scared of finding myself in situations that will lead to me being diagnosed with HIV. I’m scared of HIV and AIDS in general … due to the things I have witnessed in person, you see. Some of the things that have happened and I am very stressed, you see … I fear to know [*my*] HIV status. What will happen if I find that I am HIV-positive? It is fear, although there are people who encourage us to test for HIV, and if you test positive, you will take medication and they would say “you are alive and well. Life goes on.” There is fear, I won’t lie.’ (P4, 26 years, Steadville)

‘They [*young men*] fear for their image and reputation. They think people will talk about them saying [*that*] they saw them at the clinic. They do not trust themselves. They are fearful as men. Let us say, if I find out that I now have HIV and AIDS, how will I feel, how will people treat me, my life will change.’ (P17, 18 years, Driefontein)

‘[*T*]hey [*young men*] do not love testing for HIV, they are scared. They fear the outcome of the results.’ (P6, 21 years, Steadville)

**Sub-theme 1.2: Limited HIV and HIV-testing services knowledge:** Some of the participants believed that young men did not test for HIV because of both a lack of information on HIV, and the stigma associated with both HIV and HIV testing in their communities. This point was related in the following ways:

‘[*They*] lack knowledge. They do not have any knowledge because few get tested, and they are not interested in being educated about it either.’ (P13, 21 years, Driefontein)

‘In the rural area[*s*], guys do not take HIV testing seriously. It is not taken seriously. I think it is knowledge. There is lack of knowledge in the rural areas because of the terms we still use when referring to people who take HIV medication, such as “uyagwinya” [*meaning someone who is on ART*]. It is even difficult to disclose to someone that you take HIV medication. We still discriminate against people who live with HIV. I think that HIV knowledge has not been introduced thoroughly.’ (P11, 28 years, Driefontein)

**Sub-theme 1.3: Stigma around HIV/AIDS:** Some participants highlighted the prevalence of stigma around HIV/AIDS that exist in their communities for those who test positive. Participants noticed the following:

‘I think it’s still a taboo subject … I think it depends on what kind of guys they are because I personally hang out with different guys. Others do disclose that they live with HIV, they are on medication, and they live a normal life. And you do get other people who still perceive HIV as a taboo topic, maybe thinking people will start thinking they acquired the virus from failing to control themselves.’ (P1, 30 years, Steadville)

One participant mentioned that some young men saw HIV/AIDS as a taboo subject and thought that women would deprive them of sexual intercourse should they ever test positive for HIV. He said the following:

‘[*T*]here is still that stigma that if I have the virus, now women will not have sex with you.’ (P8, 22 years, Steadville)

#### Theme 2: Motivators to HIV-testing services utilisation

The research findings revealed that young men who accessed HTS had a variety of reasons for wanting to test for HIV.

**Sub-theme 2.1: Engaging in unsafe sexual intercourse:** Most participants stated that they decided to test for HIV following unsafe sexual intercourse with individuals they did not trust. Participants had the following to say:

‘The last [*HIV test*] was recently in January, because I slept with someone without using protection. I didn’t trust the person after we did it. It was something in my mind that, you know, this one was not okay. So, I tested and I was okay.’ (P2, 30 years, Steadville)

‘In 2018 … when I was a student at [*university name withheld*]. There was a girl that I can say I loved a lot. One night, I managed to get her … I was also coming from somewhere and we ended up going to her room and had sex under the influence of drugs, and sometimes one forgets about things that are a priority, my brother, like a condom. I had sex with her …’ (P4, 26 years, Steadville)

**Sub-theme 2.2: Undergoing voluntary medical male circumcision:** Some respondents said that they tested for HIV because they had decided to go for VMMC. Research participants said the following:

‘It was because of the circumcision … when you get there, you test for HIV and go back to fetch the results.’ (P11, 28 years, Driefontein)

‘[*A*]nd also tested when I was circumcised so I saw that it [*HIV*] is something that is tested every time you go to hospital.’ (P3, 20 years, Steadville)

‘I first went in 2016 [*to test for HIV*] when I got circumcised.’ (P13, 21 years, Driefontein)

**Sub-theme 2.3: Early HIV detection:** Participants cited early detection of HIV and encouraging others to test as important reasons for testing for HIV. They knew that it was essential to be initiated onto HIV care early, and that knowing their status ensured that they could protect others. They reported that:

‘It is important to know your status all the time, so that [*you*] can get help earlier.’ (P13, 21 years, Driefontein)

‘It is important to test for HIV because you then know if you have HIV or not. So, it is important to test to know if you are still okay or not. It helps to know that you are HIV-positive sooner, so that you will be able to start medication.’ (P12, 25 years, Steadville)

**Sub-theme 2.4: Awareness of HIV status:** Being aware of their HIV status to enable them to lead a healthy sexual lifestyle and prevent onward transmission also emerged as reasons for testing for HIV, as indicated by their responses:

‘It is important to know your status, and to get enough information on how to prevent getting it and spreading it.’ (P17, 18 years, Driefontein)

‘What I can say is that it is important for everyone to get tested, so you know your status … it’s [*not*] something you can take lightly, so best be informed and behave accordingly.’ (P16, 26 years, Driefontein)

**Sub-theme 2.5: Having a significant other living with HIV:** The findings revealed that having a significant other who lives with HIV was a trigger for young men to decide to test for HIV, as per their comments:

‘What influenced my decision to test for HIV was finding out about my friend’s status, which made me to ask myself why I was afraid because my close friend was fine, relaxed and stable emotionally.’ (P10, 26 years, Steadville)

‘[*W*]hen I fetch treatment for my significant other, they [*nurses*] do ask me to test, then I test.’ (P8, 22 years, Steadville)

Some participants indicated that other men’s reluctance to test made them think positively about their lives and get tested. They said:

‘In my life, it has been well, I saw it [*as*] important to test for HIV.’ (P17, 18 years, Driefontein)

‘Other men’s beliefs of HIV testing motivated me to test for HIV and know my status.’ (P7, 22 years, Steadville)

## Discussion

Seventeen (11 peri-urban and 6 rural) young black African IsiZulu-speaking men participated in this study, all of whom were unmarried; eight were unemployed. While all had experienced HTS, they were aware of the reasons that hindered themselves and their peers from accessing such services. The literature speaks of the difficulty of getting men to test for HIV, regardless of age,^10^ as they are known as a population that seldom utilises HTS, hence this study exploring some of the barriers and facilitators to HIV testing experienced by young isiZulu men.

The study found that fear of a positive test result and limited information on HTS, and stigma associated with being infected, inhibited young men from testing for HIV. Participants stated that they were afraid to test for HIV because of a possible positive result, with the associated consequences. They stated that the possible reasons for this fear were their unsafe sexual practices, including having sex with women that they did not trust, and fear of HIV as a disease, and weighed in on why they believe other young men do not test for HIV. These findings are similar to those of a study by Hlongwa et al., which explored the barriers to HIV-testing uptake among men in other regions of sub-Saharan Africa, and found that fear of testing for HIV was influenced by engaging in unsafe sexual practices with multiple sexual partners.^10^ There did seem to be some ambivalence among young men who were fearful that they might be infected with HIV, yet continued to engage in risky sexual activities. This ambivalence has been highlighted in studies by Mohlabane et al.^23^ and Conserve et al.,^35^ which found that men were scared to test for HIV because of the fear of testing positive, yet continued to engage in unsafe sexual practices. Fear of testing for HIV may lead to difficulty in accepting an HIV-positive diagnosis, and could potentially affect adherence to ART. Exploring this ambivalence may provide opportunities to discuss their knowledge, attitudes and practices because, as shown in this study, being knowledgeable about HIV does not automatically translate into the uptake of HTS by men, or choosing safe sexual practices.^10,23,36^

Participants further revealed that testing positive for HIV had the potential to ruin their reputation and that of other young men in the community, particularly if they are seen by people coming out of an HTS consulting room. This finding suggests the fear of being known to live with HIV by other community members, and the stigma attached to being diagnosed with HIV, were a significant constraint to the uptake of HTS. This finding is similar to a study by Sileo et al., who found that testing positive for HIV tainted men’s reputation by labelling them as sexually reckless and promiscuous.^37^ There is a lack of literature on how the stigmatising language may harm men and influence their willingness to test for HIV. Fear of testing for HIV delays health-seeking behaviour and initiation of HIV treatment and care, and risks ongoing transmission of the virus to sexual partners as well as the progression of HIV infection to AIDS.

Limited HIV, HTS knowledge and stigma emerged as other reasons for men’s lack of utilisation of HTS, which is consistent with previous research.^38,39^ Stigma delays the utilisation of HTS because of the fear of being judged and discriminated against.^22^ These findings suggest that knowledge of HIV/AIDS and HTS could be important for reducing stigma about HTS and PLHIV in their communities. Because of prevalent stigma, young men may perceive HIV and AIDS as a fatal disease, particularly if they grow up in communities where both are poorly understood and associated mortality rates are high; as a result, they may face discrimination and may be hesitant to utilise HTS. Ryan et al. maintain that PLHIV are at a high risk of being discriminated against based on their HIV-positive status compared with those whose HIV status is not known in their communities.^40^

This study revealed a generally positive consensus among the young men about the benefits of utilising HTS. They expressed a number of reasons for testing for HIV and the importance of taking such a step, with the majority citing that they had engaged in unsafe sexual practices, were curious to know their HIV status, and understood the importance of early detection of the virus should they test HIV-positive. This finding is similar to a South African study by De Wet et al. on the accuracy of HIV knowledge among youth affected by HIV. They found that despite basic but accurate HIV knowledge among most young people, they continued to engage in unsafe sexual practices, which highlights the disconnect between knowledge and practice.^36^ The findings also suggested that a lack of trust in sexual partners played an important role in getting young men to test for HIV, which may have contributed to not trusting themselves if they were HIV-negative. From the responses, it was unclear as to whether they were referring to female or male sexual partners, or both, as this was not explored.

The findings indicated that although the young men tested for HIV, they did not change their risky sexual behaviour. The plausible reasons for this could include peer pressure, and alcohol and drug use, which alter young men’s thinking processes, as well as societal beliefs that normalise risky sexual practices to prove masculinity.^41,42^ However, normalising unsafe sexual practices increases the probability of unplanned and unwanted pregnancies and contracting sexually transmitted infections.^43^ This finding is supported by Ragonese, Shand and Barker, who reported that masculinity in traditional Zulu communities is proven through engagement in sexual intercourse with multiple partners, as well as drug and alcohol use.^44^ Therefore, there is a need for effective sexual behaviour change interventions targeting young men in Ladysmith, that take into consideration their understanding of masculinity.

The findings also revealed that VMMC was one of the main reasons for accessing and utilising HTS among the participants. Currently in SA, VMMC is promoted as an effective HIV-prevention strategy, and that for a men to be circumcised at a healthcare facility, HIV testing is voluntary.^45,46^ This finding is supported by a South African study by Nxumalo and Mchunu about the relationship between VMMC and HIV prevention, where HTS was found to be an essential part of the intervention.^47^ VMMC provides an opportunity to reach out to uncircumcised young men, educate and counsel them about HIV, and contribute to a reduction in new infections among this vulnerable population.

A study by Kikaya et al. in Lesotho maintained that VMMC remained one of the pivotal strategies, not only to test men for HIV, but also to link and initiate those who tested HIV-positive to treatment and care.^48^ However, the findings of this study did not clarify whether or not there was a cultural influence for men to undergo VMMC, which is unlikely, as circumcision is not a Zulu cultural expectation. There remains a need to strengthen efforts to scale up VMMC services, particularly in hard-to-reach settings, in order to get more young men to test for HIV and be initiated into treatment and care should they test HIV-positive.^49^ This finding calls for the strengthening of the VMMC initiative, which the government has been driving for many years.^49^ It must be emphasised that there is a need to be aware of young men’s cultural and traditional beliefs, among other factors, to help facilitate their decision to undergo circumcision.

Early detection of HIV was perceived by young men as an important step towards getting an HIV-positive diagnosis to protect people or a person with whom they may be having a sexual relationship. The authors believe that these findings highlight the ambivalence among young men in that they wanted to know their HIV status, but still engaged in risky sexual behaviour, at times with people about whose HIV status they were unsure. In addition, these findings suggest that young men understood the importance of knowing their HIV status. HIV testing requires an assessment of young men’s readiness for either a positive or negative outcome, and to know how to navigate around the diagnosis, should they test positive. Acceptance of the result can lead to greater adherence, and disclosure to others,^50,51^ as well as encourage other men to test. An important finding from this study is that (many) young men recognise the need for HIV testing and were willing to test, but that the barriers of fear and stigma prevented many from doing so.

Despite engaging in unsafe sexual practices, it was not clear from the results whether the participants viewed themselves as being high risk for acquiring HIV. This is consistent with a South African study by Muravha et al. on perceptions of risk behaviour and drivers to test for HIV among youth (18−25 years), most of whom perceived themselves as being at low risk of acquiring HIV/AIDS, and were (largely) unaware of their HIV status.^52^ The authors found that testing and detection of HIV helped young people to know their HIV status and to initiate treatment when necessary.^52^ Overall, the need to test for and detect HIV plays an important role in the journey of initiating and adhering to treatment upon finding out that they are living with HIV. In terms of behaviour change, these findings concur with those of Aloni, Mbago and Sichona, who point out that early HIV detection is essential and could possibly facilitate behaviour change.^53^ An HIV-negative result could also be used to emphasise the practice of safer sex and the need to have a reduced number of sexual partners, as well as to reduce the anxiety associated with the possibility of getting an HIV-positive result when deciding to test for HIV. However, young men did not mention if they experienced any form of anxiety, or what informed their decision when they decided to test for HIV.

The findings further highlighted young men’s curiosity about knowing their HIV status when they were not already aware of it. However, after testing for HIV, some young men continued to engage in risky sexual behaviour, which necessitated additional HIV testing; this finding suggested that counselling and an HIV result did not influence their sexual behaviour. This finding is also consistent with a study by Bay et al. on knowledge of HIV and testing for it among men who have sex with men (MSM) in Brazil, which found that approximately 36% of participants tested for HIV as a result of being curious about their status.^54^ Having a significant other, be it a friend or relative, who was openly living with HIV served as another reason for young men to be willing to test for HIV, because of the trust and hope that such a person will decide to want to know their HIV status. However, there is a scarcity of literature in the African context to support this finding.

### Strengths and limitations

A strength of the study is the number of participants who were interviewed, which enabled data saturation to be reached. A limitation of qualitative research is that the findings cannot be generalised to other populations and may not be representative of all experiences of young men in Steadville and Driefontein. In Steadville, where the author is from, some research participants may have expressed what they thought the author wanted to hear, because he is from the area. The principal researcher was unable to interpret the respondents’ facial and body language and probe them because of the use of the WhatsApp communication platform and telephonic audio calls as data collection tools, which may have been a limiting factor. Also, it would have been interesting to find out more about the barriers to HTS from young men who had not accessed HTS.

## Conclusion and recommendations

Findings from this study revealed several barriers and facilitators that influence young men’s decision to test for HIV, these being important to understand to be able to attract young men to test and retain them in HIV care in both rural and peri-urban communities. There is also a need for interventions targeting men to raise awareness of HTS at community and healthcare facility level. Risk-reduction programmes, such as HTS counselling, disclosure counselling for young MLHIV, comprehensive and integrated health screening, VMMC, ART adherence clubs, and zero-stigma programmes need to be strengthened, and ongoing behaviour change programmes modified and implemented as new findings emerge, to encourage effective and sustainable behaviour change among young men. More research is needed to understand the HTS experiences of young men on a larger scale in Ladysmith and the broader KZN province, to provide a regional overview of men’s experiences. In addition, there is a need for health education that will enable and convince men to practise safer sex and prevent of the onward transmission HIV.
